# Circulating MicroRNAs for Diagnosis of Acute Pulmonary Embolism: Still a Long Way to Go

**DOI:** 10.1155/2022/4180215

**Published:** 2022-01-10

**Authors:** Matteo Sobrero, Fabrizio Montecucco, Federico Carbone

**Affiliations:** ^1^First Clinic of Internal Medicine, Department of Internal Medicine, University of Genoa, 6 Viale Benedetto XV, 16132 Genoa, Italy; ^2^IRCCS Ospedale Policlinico San Martino, Genoa-Italian Cardiovascular Network, 10 Largo Benzi, 16132 Genoa, Italy

## Abstract

Venous thromboembolism (VTE) represents the third most frequent cause of acute cardiovascular syndrome. Among VTE, acute pulmonary embolism (APE) is the most life-threatening complication. Due to the low specificity of symptoms clinical diagnosis of APE may be sometimes very difficult. Accordingly, the latest European guidelines only suggest clinical prediction tests for diagnosis of APE, eventually associated with D-dimer, a biomarker burdened by a very low specificity. A growing body of evidence is highlighting the role of miRNAs in hemostasis and thrombosis. Due to their partial inheritance and susceptibility to the environmental factors, miRNAs are increasingly described as active modifiers of the classical Virchow's triad. Clinical evidence on deep venous thrombosis reported specific miRNA signatures associated to thrombosis development, organization, recanalization, and resolution. Conversely, data of miRNA profiling as a predictor/diagnostic marker of APE are still preliminary. Here, we have summarized clinical evidence on the potential role of miRNA in diagnosis of APE. Despite some intriguing insight, miRNA assay is still far from any potential clinical application. Especially, the small sample size of cohorts likely represents the major limitation of published studies, so that extensive analysis of miRNA profiles with a machine learning approach are warranted in the next future. In addition, the cost-benefit ratio of miRNA assay still has a negative impact on their clinical application and routinely test.

## 1. Introduction

Venous thromboembolism (VTE) is a family of disease that includes deep venous thrombosis (DVT) and acute pulmonary embolism (APE). VTE is commonly found in clinical practice being the third cause of acute cardiovascular syndrome after myocardial infarction and stroke [1]. Among this class of disease, APE represents the most serious complication of VTE thus requiring early diagnosis and treatment. The incidence of APE ranges from about 39 to 115 per 100.000 population every year and accounts for about 300,000 death/year in US [2]. A recent increased in its incidence was also observed during SARS-CoV-2 outbreak, likely as an expression of the thromboinflammatory storm triggered by infection [3]. Symptoms of APE are often not specific—ranging from mild symptoms to sudden death—and clinical diagnosis may be sometimes very difficult [4]. Pulmonary angiography and computed tomography pulmonary angiography (CTPA) are highly specific for diagnosis of APE [5], but they require intravenous contrast infusion that may represents a great concern in patients with chronic kidney failure or allergic diathesis. Latest European guidelines published in 2019 have included two major prediction tests for APE diagnosis: the revised Geneva rule and the Wells score [6]. Their goal is to increase the rate of APE diagnosis, by stratifying patients across risk categories. Nevertheless, for only 65% of patients categorized at high-risk APE is finally diagnosed. The search for biomarkers able to implement the diagnostic chart—alone or combined with clinical scores—then represents an urgent clinical need. D-dimer assay offers high sensitivity but low specificity, thus limiting its application as exclusion test for diagnosis of APE [7, 8]. Here, we summarize the role of microRNAs (miRNAs) in VTE, with a special focus on APE. Great attention has also been paid to link current pathophysiological evidence with potential therapeutic implications.

## 2. miRNAs: Pathophysiological Actors and Potential Useful Biomarkers of Thromboembolism

miRNAs are noncoding RNAs around 22 nucleotides long [9] mainly involved in posttranslational messenger RNA (mRNAs) degradation [10]. miRNAs generate from gene exons—or less frequently introns—and processed into pre-miRNAs [11–13]. Once in the cytoplasm, pre-miRNAs are processed by a Dicer into mature miRNAs [14] that are released freely or within microvesicles. The way miRNAs are carried also has high relevance in their pathophysiology. Within microvesicles, miRNAs may complex and interact with other molecules, even those involved in thrombosis and homeostasis. Furthermore, microvesicle composition may provide information on the cellular source of miRNAs [15]. By targeting hundreds of genes [16], the spectrum of activity for each miRNAs broadly ranges from translation repression [17] to stimulation [18, 19], target degradation, and transcriptional/posttranscriptional gene silencing as well [20, 21]. The regulatory role of miRNAs is known since decades [22] but their applications as diagnostic/prognostic markers [23]—and even therapeutic targets [24]—have been only recently investigated. Many features would characterize miRNAs as ideal biomarkers: stability, low structural complexity, lack of postprocessing modifications, organ- and cell-specific expression, and tissue- and pathology-specific regulation [25–27]. miRNAs are also detectable in many fluids—e.g., such as serum, plasma, urine, and saliva [28]—where they regulate different biological processes [29, 30]. In light of these properties, miRNAs are being increasingly described as potential biomarkers of disease, including cardiovascular ones: heart failure, arrhythmias, coronary artery disease, myocardial fibrosis, and pulmonary arterial hypertension (PAH) [31, 32]. Although bioinformatics plays a big part in identifying putative miRNAs, a range of techniques has been developed to overcome technical challenges and simplifying miRNA profiling. Alongside quantitative PCR—characterized by high specificity and sensitivity but limited to small scale experiments—clinical application of miRNA assay relies on array or multiplex profiling, which maintain high sensitivity and specificity alongside with a straightforward data analysis. Much more is expected from the incoming development of RNA sequencing that would perform whole-genome analysis.

### 2.1. The Role of miRNA on Hemostasis and Thrombosis

A growing body of evidence has identified for miRNAs an active role of hemostasis and thrombosis. This effect may be driven by an active modulation of specific proteins: factor XI, plasminogen activator inhibitor 1 (PAI-1), protein S, fibrinogen, tissue factor, and antithrombin [33, 34]. Due to their partial inheritance expression and susceptibility to environmental factors, miRNAs may then have a dynamic role in VTE, which would encompass the whole classical Virchow's triad. Many of them have been then tested in clinical studies [35, 36], but a major role has been accounted for miR-134, miR-145, miR-195, miR-483-3p, miR-532, and miR-1233 [37]. Among them, miR-145 is specific of vascular smooth muscle cells (VSMCs) and exerts regulatory properties on tissue factor (TF) gene expression. miR-145 expression is also inversely correlated proinflammatory cytokines and lower incidence of thrombosis [38, 39]. Specific miRNA signatures have been then associated to all stage of thrombotic process from initiation to organization, recanalization, and resolution. Endothelial progenitor cells (EPCs) exert a control on these processes [40]. Their suppression results in the overexpression of proinflammatory cytokines and inhibition of VEGF functions, hallmark of thrombotic risk, and associated with miR-195. GABA type A receptor-associated protein like 1 activation has been identified as the direct target by which miR-195 exerts its detrimental functions on cell proliferation, migration, angiogenesis, and autophagy [41].

The role of miRNAs on hemostasis and thrombosis is not only limited to the coagulation cascade but also involves platelet activation and reactivity [42, 43]. Increase in platelet reactivity has been associated with the overexpression of miR-320 family as consequence of the interaction with the WIPF1 gene encoding for WAS/WASL-interacting protein [44]. miR-423-5p is another biomarkers of platelet aggregation [45]. Both miR-320 family and miR-423-5p may then raise susceptibility for VTE [46], whereas miR-1233 has been indicated as connecting signal between platelets and ECs [47].

Other miRNAs are finally implicated in vascular repair and thrombus resolution. This effect—mediated by angiogenesis and EPC proliferation/migration—involves miR-21, miR-126, miR-150, and miR-424 [48–51].

Although far from routinely clinical application, identifying those specific miRNA signatures—virtually targeting all Virchow's triad [52]—would have a rationale for VTE risk stratification [53].

## 3. miRNAs in Acute Pulmonary Embolism: Experimental Data and Clinical Evidence

Despite the epidemiological and clinical relevance, diagnosis of APE still represents an unmet clinical need. D-dimer is routinely used as biomarker of APE but it is burdened by a very low specificity [54]. Many other biomarkers tested in the last decades failed to replace or improve D-dimer performance [55–57]. Concerning miRNAs, experimental data are mainly focusing on vascular response to APE [58–63], while data on APE predictor/diagnosis are few. The lack of standardization their assay represents an additional confounder. However, all studies here considered share similar laboratory processes with differences in protocol of centrifugation (Table 1).

**Figure 1:**
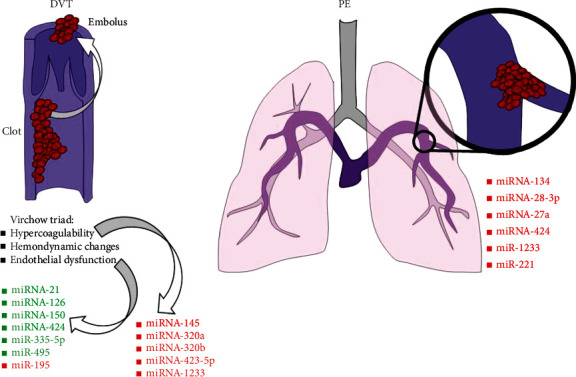
List of most important microRNAs (miRNAs) involved in venous thromboembolism. Deep venous thrombosis (DVT) determinants are classically grouped into the Virchow trial. They may be influenced by a wide range of miRNAs, especially hypercoagulability and endothelial dysfunction. Less is known about pulmonary embolism (PE). Whereas pathophysiological data are still lacking, different miRNAs are being increasingly described as potential biomarker of disease.

First in 2011, a panel of 30 different miRNAs was tested in a case control study matching patients with suspected APE [64]. Among the most expressed miRNAs (10-fold or higher), plasmatic miR-134 was the best predictor of APE, also able to identify high/intermediate *vs.* low-risk patients. miR-134 was then identified as specifically expressed by mononuclear blood cells [65] but its specificity for APE diagnosis was not later confirmed. Rather, a persistent increase (about 3.6-fold) in miR-28-3p was observed even hours after the onset of APE [66]. A release by hypoxic-ischemic lung cells as response to inositol phosphate metabolism and phosphatidylinositol pathway activation has been also hypothesized as mechanisms for the miR-28-3p expression. More recently, plasmatic concentrations of miR-27a and miR-27b emerged as further potential diagnostic markers of APE, being able to increase diagnostic potential of D-dimer [67]. Accordingly, the miR-27 family is known to regulate the TF pathway inhibitor (TFPI) in ECs [68, 69]. Platelet-derived miR-1233 is another candidate biomarker associated with suppressant activity on platelet activation and P-selectin expression [70]. Clinical relevance of miR-1233 relies on the ability in discriminating APE from other thrombotic disease (e.g., non-ST elevation myocardial infarction (NSTEMI) and DVT) with an early predictive value, higher than miRNA-27a and miRNA-134 [71]. Finally, more recent analysis on extensive miRNA panels identified a role for plasmatic miR-221, a VSMC-specific product upregulated by platelet-derived growth factor [72, 73]. Plasmatic miR-221 significantly increased in patients with APE with a cut-off set at 4-fold overexpression [74]. The abovementioned studies provide the rationale but lacks of definitive proof for translating miRNA assay in clinical practice (Figure 1). Small sample size and bias involving patient selection and miRNA analysis protocol are likely their main weakness. Future studies are expected to address these issues performing real-life analyses and finally test whether miRNA assay may overcome D-dimer in diagnosing APE. To date, miRNA assay still remains far from any potential clinical translation.

## 4. Conclusion

The recognition of a circulating biomarker able to stratify the risk for APE would lead more accurately patients with clinical suspect of APE to second-level diagnostic tools, such as CTPA. This would be of paramount relevance for patients with mild/severe contraindications to CTPA, especially those with chronic kidney disease or history of medium contrast allergic reactions. Data from preliminary studies also suggest a potential role of miRNAs in discriminating APE from the most frequent confusing clinical conditions (firstly NSTEMI). Such an achievement would shorten the diagnostic chart and the time-to-treatment protocols, in accordance with the classic paradigm of “golden hour.” Even if it were, benefit of miRNA assay should also exceed the economic burden of their assay. The need of large panel assays and interaction analyses makes machine learning approach mandatory to finally establish the sensitivity of miRNA (as single biomarker or panel) and their clinical relevance. Additional goals should also include high sensitivity and specificity for APE. Comparing miRNAs with other biomarkers of APE (e.g., NT-proBNP, troponins, and D-dimer) would further help in establishing their potential for clinical use. Not last, being miRNA expression partially inherited, large-scale studies should consider different ethnic groups. Finally, any potential role of anticoagulation therapies on miRNA expression remains unclear.

## Figures and Tables

**Table 1:** miRNAs studied as potential diagnostic biomarkers for APE.

Author	Year	Study design	miRNA (cut-off)	Sample	Results	Concerning
Mao et al. [63]	2011	32 APE patients vs. 32 healthy controls vs. 22 non-APE patients^∗^	miRNA-134 (10-fold difference between miRNA levels)	Plasma	miRNA-134 was significantly higher in APE with an AUC of 0.83 (95% CI, 0.74 to 0.93) *p* < 0.001	miRNA-134 was elevated also in UA
Hoekstra et al. [65]	2016	37 APE patients vs. 37 healthy controls	miRNA-28-3p (4-fold difference between miRNA levels)	Plasma	miRNA-28-3p (but neither miRNA-134 nor miRNA-210) show a significant increase—stable during the first 6 hours—in APE. The AUC was 0.79 (95% CI 0.69 to 0.90)	miRNA-28-3p was elevated also in DM and GI malignancies
Zhou et al. [66]	2018	78 APE patients vs. 70 healthy controls	miRNA-27a/b	Plasma	miRNA-27a expression was upregulated in APE patients (*p* < 0.001). The AUC was 0.78 (95% CI 0.69 to 0.88); *p* < 0.001. miRNA-27a significantly improved the AUC of D-dimer	miRNA-27 levels are also influenced by LVH
Ba et al. [69]	2016	30 APE patients vs. NSTEMI (*n* = 30), DVT (*n* = 6), PAH (*n* = 15), and 12 healthy controls	miRNA-1233 (11-fold difference between miRNA levels)	Serum	In acute state (1^st^ day), miRNA-1233 was even able to discriminate APE from NSTEMI with an AUC of 0.95 (95% CI 0.89 to 1.00); *p* < 0.001 highest serum level on 1st day. miRNA-1233 then decreased levels on 3^rd^ and 5^th^ day with lower values reached at 9 months	None. Even, miRNA-1233 was better than miRNA-134 and miRNA-27a as APE biomarker
Nie et al. [72]	2018	60 APE vs. 50 healthy controls	miRNA-221 (4-fold difference between miRNA levels)	Plasma	miR-221 was significantly upregulated in APE (*p* < 0.05) and showed positive correlations with BNP, troponin, and D-dimer. AUC for plasma miR-221 was 0.82 (95% CI 0.76 to 0.91), higher than that of D-dimer	miRNA-221 was elevated also in MI and PAH

APE: acute pulmonary embolism; AUC: area under the curve; UA: unstable angina; DM: diabetes mellitus; GI: gastrointestinal; LVH: left ventricular hypertrophy; NSTEMI: non-ST elevated nyocardial infarction; DVT: deep venous thrombosis; PAH: pulmonary arterial hypertension; BNP: B-type natriuretic peptide; MI: myocardial infarction.

## References

[1] (2014). Thrombosis: a major contributor to the global disease burden. *Journal of Thrombosis and Haemostasis*.

[2] Wendelboe A. M., Raskob G. E. (2016). Global burden of thrombosis: epidemiologic aspects. *Circulation Research*.

[3] Mondal S., Quintili A. L., Karamchandani K., Bose S. (2020). Thromboembolic disease in COVID-19 patients: a brief narrative review. *Journal of Intensive Care*.

[4] Stein P. D., Beemath A., Matta F. (2007). Clinical characteristics of patients with acute pulmonary embolism: data from PIOPED II. *The American Journal of Medicine*.

[5] Konstantinides S. V., Meyer G., Becattini C. (2020). 2019 ESC guidelines for the diagnosis and management of acute pulmonary embolism developed in collaboration with the European Respiratory Society (ERS). *European Heart Journal*.

[6] Konstantinides S. V., Meyer G. (2019). The 2019 ESC guidelines on the diagnosis and management of acute pulmonary embolism. *European Heart Journal*.

[7] Stein P. D., Hull R. D., Patel K. C. (2004). D-dimer for the exclusion of acute venous thrombosis and pulmonary embolism: a systematic review. *Annals of Internal Medicine*.

[8] Douma R. A., Kamphuisen P. W., Buller H. R. (2010). Acute pulmonary embolism. Part 1: epidemiology and diagnosis. *Nature Reviews. Cardiology*.

[9] Lau N. C., Lim L. P., Weinstein E. G., Bartel D. P. (2001). An abundant class of tiny RNAs with probable regulatory roles in Caenorhabditis elegans. *Science*.

[10] Bartel D. P. (2004). MicroRNAs: genomics, biogenesis, mechanism, and function. *Cell*.

[11] Bushati N., Cohen S. M. (2007). MicroRNA functions. *Annual Review of Cell and Developmental Biology*.

[12] Cai B., Li J., Wang J. (2012). MicroRNA-124 regulates cardiomyocyte differentiation of bone marrow-derived mesenchymal stem cells via targeting STAT3 signaling. *Stem Cells*.

[13] Lee Y., Kim M., Han J. (2004). MicroRNA genes are transcribed by RNA polymerase II. *The EMBO Journal*.

[14] Ruby J. G., Jan C. H., Bartel D. P. (2007). Intronic microRNA precursors that bypass Drosha processing. *Nature*.

[15] Zifkos K., Dubois C., Schafer K. (2021). Extracellular vesicles and thrombosis: update on the clinical and experimental evidence. *International Journal of Molecular Sciences*.

[16] Gangaraju V. K., Lin H. (2009). MicroRNAs: key regulators of stem cells. *Nature Reviews. Molecular Cell Biology*.

[17] Brennecke J., Stark A., Russell R. B., Cohen S. M. (2005). Principles of microRNA-target recognition. *PLoS Biology*.

[18] Vasudevan S., Tong Y., Steitz J. A. (2007). Switching from repression to activation: microRNAs can up-regulate translation. *Science*.

[19] Kozak M. (2008). Faulty old ideas about translational regulation paved the way for current confusion about how microRNAs function. *Gene*.

[20] Hwang H. W., Wentzel E. A., Mendell J. T. (2007). A hexanucleotide element directs microRNA nuclear import. *Science*.

[21] Kim D. H., Saetrom P., Snove O., Rossi J. J. (2008). MicroRNA-directed transcriptional gene silencing in mammalian cells. *Proceedings of the National Academy of Sciences of the United States of America*.

[22] Lagos-Quintana M., Rauhut R., Lendeckel W., Tuschl T. (2001). Identification of novel genes coding for small expressed RNAs. *Science*.

[23] Reid G., Kirschner M. B., van Zandwijk N. (2011). Circulating microRNAs: association with disease and potential use as biomarkers. *Critical Reviews in Oncology/Hematology*.

[24] Elbashir S. M., Lendeckel W., Tuschl T. (2001). RNA interference is mediated by 21- and 22-nucleotide RNAs. *Genes & Development*.

[25] van Rooij E. (2011). The art of microRNA research. *Circulation Research*.

[26] Mitchell P. S., Parkin R. K., Kroh E. M. (2008). Circulating microRNAs as stable blood-based markers for cancer detection. *Proceedings of the National Academy of Sciences of the United States of America*.

[27] Wang K., Zhang S., Marzolf B. (2009). Circulating microRNAs, potential biomarkers for drug-induced liver injury. *Proceedings of the National Academy of Sciences of the United States of America*.

[28] Weber J. A., Baxter D. H., Zhang S. (2010). The microRNA spectrum in 12 body fluids. *Clinical Chemistry*.

[29] Xu P., Guo M., Hay B. A. (2004). MicroRNAs and the regulation of cell death. *Trends in Genetics*.

[30] Suarez Y., Sessa W. C. (2009). MicroRNAs as novel regulators of angiogenesis. *Circulation Research*.

[31] Kalozoumi G., Yacoub M., Sanoudou D. (2014). MicroRNAs in heart failure: small molecules with major impact. *Glob Cardiol Sci Pract*.

[32] Fichtlscherer S., de Rosa S., Fox H. (2010). Circulating microRNAs in patients with coronary artery disease. *Circulation Research*.

[33] Teruel R., Corral J., Perez-Andreu V., Martinez-Martinez I., Vicente V., Martinez C. (2011). Potential role of miRNAs in developmental haemostasis. *PLoS One*.

[34] Teruel-Montoya R., Rosendaal F. R., Martinez C. (2015). MicroRNAs in hemostasis. *Journal of Thrombosis and Haemostasis*.

[35] Starikova I., Jamaly S., Sorrentino A. (2015). Differential expression of plasma miRNAs in patients with unprovoked venous thromboembolism and healthy control individuals. *Thrombosis Research*.

[36] Wang X., Sundquist K., Svensson P. J. (2019). Association of recurrent venous thromboembolism and circulating microRNAs. *Clinical Epigenetics*.

[37] Xiang Q., Zhang H. X., Wang Z. (2019). The predictive value of circulating microRNAs for venous thromboembolism diagnosis: a systematic review and diagnostic meta-analysis. *Thrombosis Research*.

[38] O'Leary L., Sevinc K., Papazoglou I. M. (2016). Airway smooth muscle inflammation is regulated by microRNA-145 in COPD. *FEBS Letters*.

[39] Sahu A., Jha P. K., Prabhakar A. (2017). MicroRNA-145 impedes thrombus formation _via_ targeting tissue factor in venous thrombosis. *eBioMedicine*.

[40] Abou-Saleh H., Yacoub D., Théorêt J. F̧. (2009). Endothelial progenitor cells bind and inhibit platelet function and thrombus formation. *Circulation*.

[41] Mo J., Zhang D., Yang R. (2016). MicroRNA-195 regulates proliferation, migration, angiogenesis and autophagy of endothelial progenitor cells by targeting GABARAPL1. *Bioscience Reports*.

[42] Garcia A., Dunoyer-Geindre S., Fish R. J., Neerman-Arbez M., Reny J. L., Fontana P. (2021). Methods to investigate miRNA function: focus on platelet reactivity. *Thrombosis and Haemostasis*.

[43] Elia E., Montecucco F., Portincasa P., Sahebkar A., Mollazadeh H., Carbone F. (2019). Update on pathological platelet activation in coronary thrombosis. *Journal of Cellular Physiology*.

[44] Nagalla S., Shaw C., Kong X. (2011). Platelet microRNA-mRNA coexpression profiles correlate with platelet reactivity. *Blood*.

[45] Gidlof O., van der Brug M., Ohman J. (2013). Platelets activated during myocardial infarction release functional miRNA, which can be taken up by endothelial cells and regulate ICAM1 expression. *Blood*.

[46] Braekkan S. K., Mathiesen E. B., Njolstad I., Wilsgaard T., Stormer J., Hansen J. B. (2010). Mean platelet volume is a risk factor for venous thromboembolism: the Tromsø study. *Research and Practice in Thrombosis and Haemostasis*.

[47] Li J., Tan M., Xiang Q., Zhou Z., Yan H. (2017). Thrombin-activated platelet-derived exosomes regulate endothelial cell expression of ICAM-1 via microRNA-223 during the thrombosis-inflammation response. *Thrombosis Research*.

[48] Meng Q., Wang W., Yu X. (2015). Upregulation of microRNA-126 contributes to endothelial progenitor cell function in deep vein thrombosis via its target PIK3R2. *Journal of Cellular Biochemistry*.

[49] Du X., Hong L., Sun L. (2019). miR-21 induces endothelial progenitor cells proliferation and angiogenesis via targeting FASLG and is a potential prognostic marker in deep venous thrombosis. *Journal of Translational Medicine*.

[50] Bao C. X., Zhang D. X., Wang N. N., Zhu X. K., Zhao Q., Sun X. L. (2018). Retracted: MicroRNA-335-5p suppresses lower extremity deep venous thrombosis by targeted inhibition of PAI-1 via the TLR4 signalingpathway. *Journal of Cellular Biochemistry*.

[51] Hembrom A. A., Srivastava S., Garg I., Kumar B. (2020). MicroRNAs in venous thrombo-embolism. *Clinica Chimica Acta*.

[52] Fazzalari A., Basadonna G., Kucukural A. (2021). A translational model for venous thromboembolism: microRNA expression in hibernating black bears. *The Journal of Surgical Research*.

[53] Forrest A. R., Kanamori-Katayama M., Tomaru Y. (2010). Induction of microRNAs, miR-155, miR-222, miR-424 and miR-503, promotes monocytic differentiation through combinatorial regulation. *Leukemia*.

[54] Oi M., Yamashita Y., Toyofuku M. (2020). D-dimer levels at diagnosis and long-term clinical outcomes in venous thromboembolism: from the COMMAND VTE registry. *Journal of Thrombosis and Thrombolysis*.

[55] Newman J., Brailovsky Y., Allen S. (2021). Angiopoietin-2 correlates with pulmonary embolism severity, right ventricular dysfunction, and intensive care unit admission. *Vascular Medicine*.

[56] Thoreau B., Galland J., Delrue M. (2021). D-dimer level and neutrophils count as predictive and prognostic factors of pulmonary embolism in severe non-ICU COVID-19 patients. *Viruses*.

[57] Bontekoe E., Brailovsky Y., Hoppensteadt D. (2021). Upregulation of inflammatory cytokines in pulmonary embolism using biochip-array profiling. *Clinical and Applied Thrombosis/Hemostasis*.

[58] Wang M., Gu S., Liu Y. (2019). miRNA-PDGFRB/HIF1A-lncRNA CTEPHA1 network plays important roles in the mechanism of chronic thromboembolic pulmonary hypertension. *International Heart Journal*.

[59] Miao R., Gong J., Zhang C. (2020). Hsa_circ_0046159 is involved in the development of chronic thromboembolic pulmonary hypertension. *Journal of Thrombosis and Thrombolysis*.

[60] Chen H., Ma Q., Zhang J., Meng Y., Pan L., Tian H. (2020). miR‑106b‑5p modulates acute pulmonary embolism via NOR1 in pulmonary artery smooth muscle cells. *International Journal of Molecular Medicine*.

[61] Liu T. W., Liu F., Kang J. (2020). Let-7b-5p is involved in the response of endoplasmic reticulum stress in acute pulmonary embolism through upregulating the expression of stress-associated endoplasmic reticulum protein 1. *IUBMB Life*.

[62] Ou M., Zhang C., Chen J., Zhao S., Cui S., Tu J. (2020). Overexpression of microRNA-340-5p inhibits pulmonary arterial hypertension induced by APE by downregulating IL-1*β* and IL-6. *Mol Ther Nucleic Acids*.

[63] Mao H. Y., Liu L. N., Hu Y. M. (2020). Mesenchymal stem cells-derived exosomal miRNA-28-3p promotes apoptosis of pulmonary endothelial cells in pulmonary embolism. *European Review for Medical and Pharmacological Sciences*.

[64] Xiao J., Jing Z. C., Ellinor P. T. (2011). MicroRNA-134 as a potential plasma biomarker for the diagnosis of acute pulmonary embolism. *Journal of Translational Medicine*.

[65] Hoekstra M., van der Lans C. A., Halvorsen B. (2010). The peripheral blood mononuclear cell microRNA signature of coronary artery disease. *Biochemical and Biophysical Research Communications*.

[66] Zhou X., Wen W., Shan X. (2016). miR-28-3p as a potential plasma marker in diagnosis of pulmonary embolism. *Thrombosis Research*.

[67] Wang Q., Ma J., Jiang Z., Wu F., Ping J., Ming L. (2018). Diagnostic value of circulating microRNA-27a/b in patients with acute pulmonary embolism. *International Angiology*.

[68] Ali H. O., Arroyo A. B., Gonzalez-Conejero R. (2016). The role of microRNA-27a/b and microRNA-494 in estrogen-mediated downregulation of tissue factor pathway inhibitor *α*. *Journal of Thrombosis and Haemostasis*.

[69] Arroyo A., Salloum-Asfar S., Pérez-Sánchez C. (2017). Regulation of TFPI*α* expression by miR-27a/b-3p in human endothelial cells under normal conditions and in response to androgens. *Scientific Reports*.

[70] Lee B. K., Kim M. H., Lee S. Y., Son S. J., Hong C. H., Jung Y. S. (2020). Downregulated platelet miR-1233-5p in patients with Alzheimer's pathologic change with mild cognitive impairment is associated with A*β*-induced platelet activation via P-selectin. *Journal of Clinical Medicine*.

[71] Kessler T., Erdmann J., Vilne B. (2016). Serum microRNA-1233 is a specific biomarker for diagnosing acute pulmonary embolism. *Journal of Translational Medicine*.

[72] Nie X., Chen Y., Tan J. (2019). MicroRNA-221-3p promotes pulmonary artery smooth muscle cells proliferation by targeting AXIN2 during pulmonary arterial hypertension. *Vascular Pharmacology*.

[73] Davis B. N., Hilyard A. C., Nguyen P. H., Lagna G., Hata A. (2009). Induction of microRNA-221 by platelet-derived growth factor signaling is critical for modulation of vascular smooth muscle phenotype. *The Journal of Biological Chemistry*.

[74] Liu T., Kang J., Liu F. (2018). Plasma levels of microRNA-221 (miR-221) are increased in patients with acute pulmonary embolism. *Medical Science Monitor*.

